# Immunophenotyping of cerebrospinal fluid cells by Chipcytometry

**DOI:** 10.1186/s12974-018-1176-7

**Published:** 2018-05-25

**Authors:** Martin W. Hümmert, Sascha Alvermann, Stefan Gingele, Catharina C. Gross, Heinz Wiendl, Anja Mirenska, Christian Hennig, Martin Stangel

**Affiliations:** 1 Department of Neurology and Department of Clinical Neuroimmunology and Neurochemistry, Hannover Medical School, Carl-Neuberg-Str. 1, 30625 Hannover, Germany; 2 Department of Neurology, University Hospital Münster, Münster, Germany; 3 Zellkraftwerk GmbH, Hannover, Germany

**Keywords:** Immunophenotype, CSF, Cerebrospinal fluid, Flow cytometry, Slide-based cytometry, Chipcytometry

## Abstract

**Background:**

The gold standard in cerebrospinal fluid (CSF) cell immunophenotyping is flow cytometry. Nevertheless, the small amount of CSF cells and the invasive character of lumbar puncture limit the spectrum of possible investigation. Chipcytometry, a modified approach to slide-based cytometry, might be a useful tool for CSF analysis due to the possibility of iterative staining, imaging, and bleaching cycles. The aim of this study was to compare flow cytometric leukocyte subset analysis with Chipcytometry comparing the percentage distribution of distinct cell populations and the T-cell CD4:CD8 ratio. Moreover, this study investigated the interpretability of chips loaded with CSF cells and examined the applicability of Chipcytometry in clinical practice.

**Methods:**

375 CSF samples from 364 patients were analyzed by Chipcytometry using an automated upright microscope. Cell surface molecules were stained using fluorescence-labeled monoclonal antibodies. For cross-validation experiments, flow cytometry data of six patients were analyzed and matched with Chipcytometry data.

**Results:**

Our experiments showed a better agreement examined by Bland-Altman analysis for samples with CSF pleocytosis than for normocellular CSF samples. Data were more consistent for B cells and CD4:CD8 ratio than for T cells and monocytes. Advantages of Chipcytometry compared to flow cytometry are that cells once fixated can be analyzed for up to 20 months with additional markers at any time. The clinical application of Chipcytometry is demonstrated by two illustrative case reports. However, the low amount of CSF cells limits the analysis of normocellular CSF samples, as in our cohort only 11.7% of respectively loaded chips had sufficient cell density for further investigation compared to 59.8% of all chips loaded with samples with elevated cell counts (≥ 5/μl). Varying centrifuge settings, tube materials and resuspension technique were not able to increase the cell yield.

**Conclusion:**

In summary, the results demonstrate the great potential of Chipcytometry of CSF cells for both scientific questions and routine diagnostic. A new chip design optimized to meet the requirements of CSF would greatly enhance the value of this method. Cross-validation results need to be confirmed in a larger cohort.

## Background

Immunophenotyping of cerebrospinal fluid (CSF) cells by flow cytometry has helped to elucidate the pathophysiology of inflammatory neurological disorders such as multiple sclerosis (MS) [1]. Furthermore, knowledge of the immune cell composition in the CSF can have prognostic value and might therefore be useful to assist treatment decisions [2]. However, although CSF is collected as part of the diagnostic work-up in many neurological conditions, immunophenotyping of CSF cells by flow cytometry is rarely used in clinical routine. This is due to a number of limitations that arise when dealing with CSF cell samples. First, CSF withdrawal by lumbar puncture is an invasive procedure that is not without side effects and is perceived as painful by most patients. Therefore it is performed only once during the diagnostic work-up in most cases. Unfortunately, flow cytometry does not allow for storing and repeated analysis of samples. As it relies on a predefined set of markers that has to be established before the sample is processed, flow cytometry cannot be easily adjusted if new clinical questions or hypotheses arise during the analysis. In addition, the cellular content of CSF is very low compared to peripheral blood, making it difficult to obtain sufficient cell numbers in paucicellular samples for detailed analysis. Furthermore, cell viability in CSF ex vivo is poor leading to a rapid decline in cell numbers after sample collection [3].

To exploit the full potential of CSF analysis for diagnostic and scientific purposes, a new method for immunophenotyping of CSF cells is highly desirable. Ideally, it should require only small amounts of cells and be available within a short time after sample collection in order to meet the constraints of low cellularity and poor cell viability. Furthermore, it should have the potential for storing and reanalyzing samples so that maximum information can be gained from a single sample and repeated lumbar punctures are unnecessary even if new questions are to be addressed.

Slide-based cytometry was first introduced in 1997 as an alternative to flow cytometry [4]. Briefly, this method is based on automated epifluorescence microscopy of cells that are immobilized on a solid surface. Since then, this concept has been refined in order to meet the requirements for peripheral blood cell analysis [5, 6] with iterative restaining [7] and photobleaching of fluorochromes [8] as newly introduced components. Chipcytometry is a modified approach to slide-based cytometry developed at the Hannover Medical School. It is based on microfluidic chips containing cell-adhesive surfaces that allow for quick and easy sample preparation, long-term storage, and repeated staining and washing steps by simple fluid exchange [9]. Through iterative staining-imaging-bleaching cycles, a theoretically unlimited number of cellular markers can be analyzed in a single sample.

So far, Chipcytometry has mostly been applied to study human peripheral blood mononuclear cells (PBMC) [10, 11] and bronchoalveolar lavage cells [12]. We aimed to investigate whether Chipcytometry is a suitable method for immunophenotyping of CSF cells.

## Methods

### Patients and sample collection

Patients were recruited at the Department of Neurology at Hannover Medical School, Germany, and the Department of Neurology at the University of Münster, Germany. The study was approved by the local ethics committee (no. 1322-2012) and all patients gave written consent before enrollment. In total, 375 CSF samples (Hannover: 364, Münster: 11) were collected from 364 patients (Hannover: 353, Münster: 11) between March 2012 and February 2016 (Table 1). Lumbar punctures were part of routine diagnostics or, in the case of two patients, were performed repeatedly for intrathecal drug injections. In addition to the routine procedures up to 5 ml of CSF were collected for study purposes. In some cases where normal pressure hydrocephalus was suspected, large volumes of CSF were withdrawn and up to 15 ml were used for the study.

**Table 1:** Number of CSF samples related to group of diseases, patient age and sex

Group of diseases	Patients *n*	Age, years Range (median)	Sex f:m (percentage)
All	364	17–85 (51)	178:186 (49:51)
MS/CIS	76	17–78 (41)	50:26 (66:34)
OIND - infectious	12	27–78 (46)	8:4 (67:33)
OIND - autoimmune	54	18–79 (48)	29:25 (54:46)
Tumor	18	28–84 (64,5)	5:13 (28:72)
NIND	167	20–85 (60)	69:98 (41:59)
NND	35	18–81 (41)	17:18 (49:51)
Unspecified	2	20, 55	0:2 (0:100)

*MS* multiple sclerosis, *CIS* clinical isolated syndrome, *OIND* other inflammatory neurological disease, *NIND* non-inflammatory neurological disease, *NND* non-neurological disease

### Sample preparation

CSF was collected in 15 ml conical bottom tubes. For study purposes, we used tubes from two different materials (polypropylene, Greiner Bio-One, Austria; or polystyrene, Sarstedt, Germany). After lumbar puncture, CSF leukocytes and erythrocytes were immediately counted in a Fuchs-Rosenthal chamber. Cells were separated from CSF by centrifugation (10 min, Centrifuge 5810R, Eppendorf, Germany). For comparison of cell yield centrifuge settings varied (G: 140, 1000, or 2400×*g*; temperature: 4 or 20 °C). The cell-free supernatant was carefully withdrawn with a pipette, leaving a volume of no more than 50 μl that was used for resuspension of the cell pellet. The suspension was then pipetted into a microfluidic chip containing a glass surface covered with oligonucleotides for non-selective cell binding. After an incubation time of 10 min at room temperature, the chip’s microfluidic channel was filled with 4% phosphate-buffered formaldehyde (Carl Roth, Germany) and stored at 4 °C for 15 min. Then, the channel was rinsed with phosphate-buffered saline (PBS). Afterwards, the chip was ready for staining with fluorescent dyes or for long-term storage at 4 °C.

### Staining and data acquisition

**Fig. 1:**
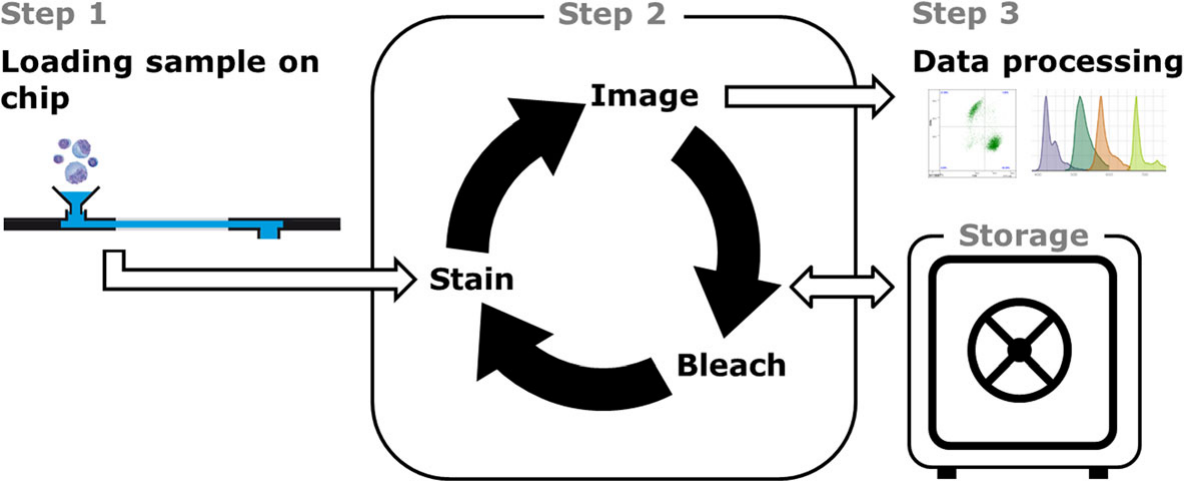
Schematic diagram of the sequential analysis by imaging-based Chipcytometry

For data acquisition with Chipcytometry, samples were processed as previously described [9]. Briefly, the method is based on iterative cycles of staining, imaging, and bleaching (Fig. 1). Each cellular biomarker was assessed separately, and no multicolor staining was used in order to avoid the need for compensation steps. Cell surface molecules were stained using phycoerythrin (PE)-labeled monoclonal antibodies diluted in PBS as indicated in Table 2 (A). For staining, the microfluidic channel was filled with the respective antibody solution and incubated for 5 min at room temperature. Then, the chip was rinsed with PBS and applied to the imaging system. For imaging, an automated upright microscope (modified Axio Observer Z1, Zeiss, Germany) was used as detailed previously [9]. For every position, two images (fluorescence light mode and transmitted light mode) were acquired. Afterwards, the remaining fluorescence was bleached by extended illumination to allow for staining with a new antibody.

**Table 2:** Antibodies used for (A) Chipcytometry and (B) flow cytometry

Epitope	Clone	Company	Dilution
A			
CD3	UCHT1	BD	1:100
CD4	RPA-T4	Biolegend	1:100
CD8	RPA-T8	BD	1:30
CD14	RMO52	Beckman Coulter	1:30
CD16	3G8	Biolegend	1:100
CD19	HIB 19	eBioscience	1:100
CD24	ML5	Biolegend	1:300
CD25	M-A251	BD	1:50
CD27	LG.3A10	Biolegend	1:300
CD38	HB7	BD	1:50
CD45RA	HI100	Biolegend	1:400
CD54	HA58	BD	1:30
CD56	AF12-7H3	Miltenyi Biotec	1:30
HLA-DR	G46-6	BD	1:50
IgD	IA6-2	Biolegend	1:30
IgM	MHM-88	Biolegend	1:250
IgG	IS11-3B2.2.3	Miltenyi Biotec	1:100
IgA	IS11-8E10	Miltenyi Biotec	1:200
Kappa light chain	MHK-49	Biolegend	1:200
Lambda light chain	MHL-38	Biolegend	1:200
B			
CD3 (PC 5.5)	UCHT1	Beckman Coulter	1:200
CD4 (APC)	12B8.2	Beckman Coulter	1:200
CD8 (pacific blue)	B9.11	Beckman Coulter	1:200
CD14 (FITC)	RM052	Beckman Coulter	1:200
CD19 (APC-A700)	J3-119	Beckman Coulter	1:200
CD45 (krome orange)	J.33	Beckman Coulter	1:200

All antibodies used for Chipcytometry were labeled with the fluorochrome phycoerythrin (PE). The fluorochromes used in flow cytometry are indicated in parentheses

### Data analysis

Cells were distinguished from the background in transmitted light mode images by automated cell recognition [9]. However, in most cases manual corrections were necessary as some cells were missed by the algorithm and sometimes debris and foreign particles were falsely marked as cells. The mean fluorescence intensity (MFI) per cell was measured before and after each bleaching procedure. The MFI after bleaching was subtracted from the MFI of the unbleached cell to account for autofluorescence and possible local inhomogeneity of illumination. Size, coordinates on the chip, and fluorescence intensities were recorded for each cell. For further analysis, data sets were processed using an in-house software. Data were visualized either in two-dimensional (2D) plot form or as heatmap images. We used 2D plots to study cell populations in a hypothesis-based approach according to predefined gating strategies. Heatmap images were generated by cluster analysis where cells with similar biomarker expression were grouped automatically, allowing for a more exploratory approach.

### Cross-validation by flow cytometry

For cross-validation experiments flow cytometry data were acquired and analyzed using a Navios™ flow cytometer and Navios™ analysis software (Beckman Coulter, USA) as previously described [13]. Briefly, 3 ml of CSF were collected in polypropylene tubes and processed within 20 min after lumbar puncture. Cells were obtained from CSF by centrifugation (15 min, 290×*g*, 4 °C). For erythrocyte lysis, the cell pellet was resuspended in 1 ml VersaLyse buffer (Beckman Coulter, Germany) and was incubated for 10 min at room temperature. Cells were stained using fluorochrome-conjugated antibodies as indicated in Table 2 (B).

### Statistics

Results were analyzed with GraphPad Prism 5.02 (GraphPad Software, USA). Statistical significance was evaluated by unpaired *t* test or analysis of variance and the use of Bonferroni’s correction. *P* values < 0.05 were considered as statistically significant. For cross-validation experiments, data sets from different methods (flow cytometry and Chipcytometry) were compared using Bland-Altman analysis.

## Results

### Cell counts and density

Out of 375 CSF samples, 283 (75.5%) had normal cell counts < 5/μl and 92 (24.5%) had a pleocytosis (≥ 5/μl). In 131 samples (34.9%) absolute cell numbers (i.e., cell concentration multiplied by volume) were ≥ 10,000.

After preparing a chip with a cell sample, cell density on the chip surface was first assessed visually using an upright microscope. Low cell density would make it unlikely that sufficient cell numbers would be recorded for statistical analysis. Based on preliminary experiments, we decided on a cut-off value of at least 20 cells per field of view. If cell density was lower than that, the chip was discarded for further analysis. Only 11.7% of chips loaded with normocellular samples had sufficient cell density and were analyzed further. Samples with elevated cell counts ≥ 5/μl produced better results, as 59.8% of the chips could be analyzed. Of the samples with absolute cell numbers < 10,000 only 9% achieved a sufficient cell density, as opposed to 50.4% of samples with ≥ 10,000 cells (Table 3). For the comparison of methods, 11 samples from different patients, 2 of them with pleocytosis, were collected at the Department of Neurology at the University of Münster and were analyzed by flow cytometry on-site. For these samples, cell density for analysis by Chipcytometry was enhanced using a minimum of 3 ml of CSF. Sufficient cell density was reached in 6 patients (54.5%).

**Table 3:** Numbers and percentages of chips with sufficient cell density subject to cell content of the CSF sample

	*n*	Chips with sufficient cell density
Cell count per μl		
< 5	283	33 (11.7%)
≥ 5	92	55 (59.8%)
≥ 10	57	37 (64.9%)
≥ 30	25	20 (80%)
≥ 50	13	11 (84.6%)
Absolute cell count		
< 10,000	244	22 (9%)
≥ 10,000	131	66 (50.4%)
≥ 50,000	39	27 (69.2%)
≥ 100,000	22	16 (72.7%)
All samples	375	88 (23.5%)

### Cell loss due to centrifugation

**Fig. 2:**
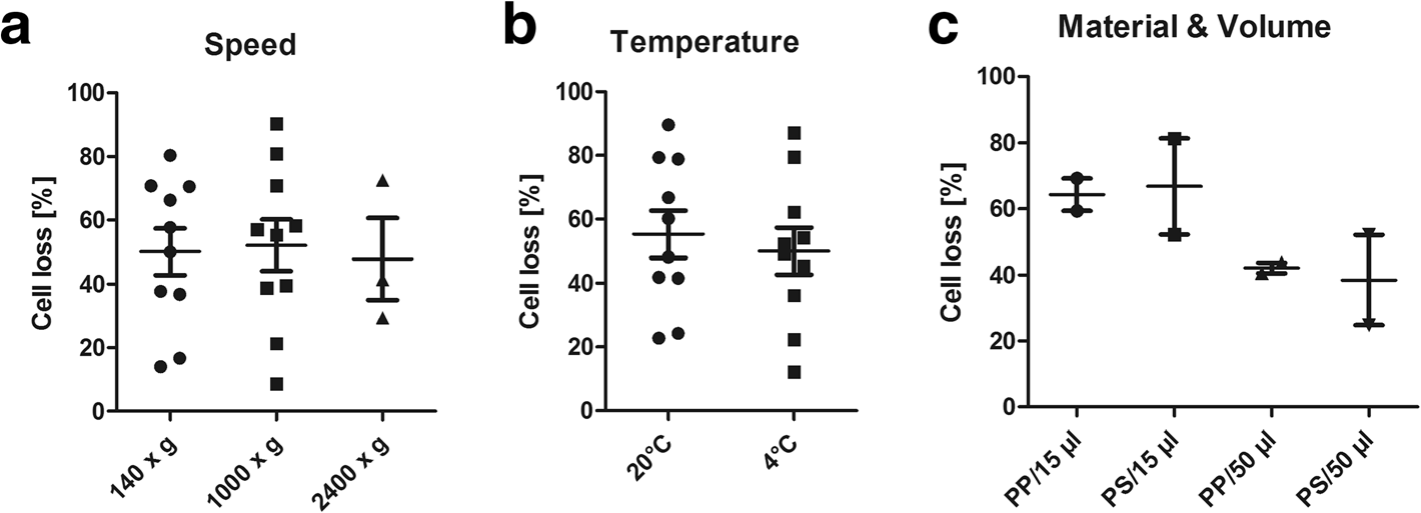
Influence of centrifuge speed (**a**), centrifuge temperature (**b**), collection tube material (**c**), and resuspension volume (**c**) on cell loss during centrifugation. PP = polypropylene, PS = polystyrene

The low cell densities on many chips led us to investigate cell loss during centrifugation. In a series of 11 CSF samples, absolute cell numbers were calculated before and after centrifugation (1000×*g*; 20 °C; 10 min). We observed a mean cell loss of 59.3% (data not shown). We aimed to minimize cell loss by modifying centrifuge settings. However, neither varying centrifuge speed nor temperature led to a significant improvement. We next evaluated whether different materials of collection tubes or different resuspension techniques had an influence on cell recovery. We found no difference between polypropylene and polystyrene tubes but resuspending cells in a larger volume (50 vs. 15 μl) after centrifugation led to a slightly increased cell yield. However, the results did not reach statistical significance (Fig. 2).

### Stability of cells and biomarkers

**Fig. 3:**
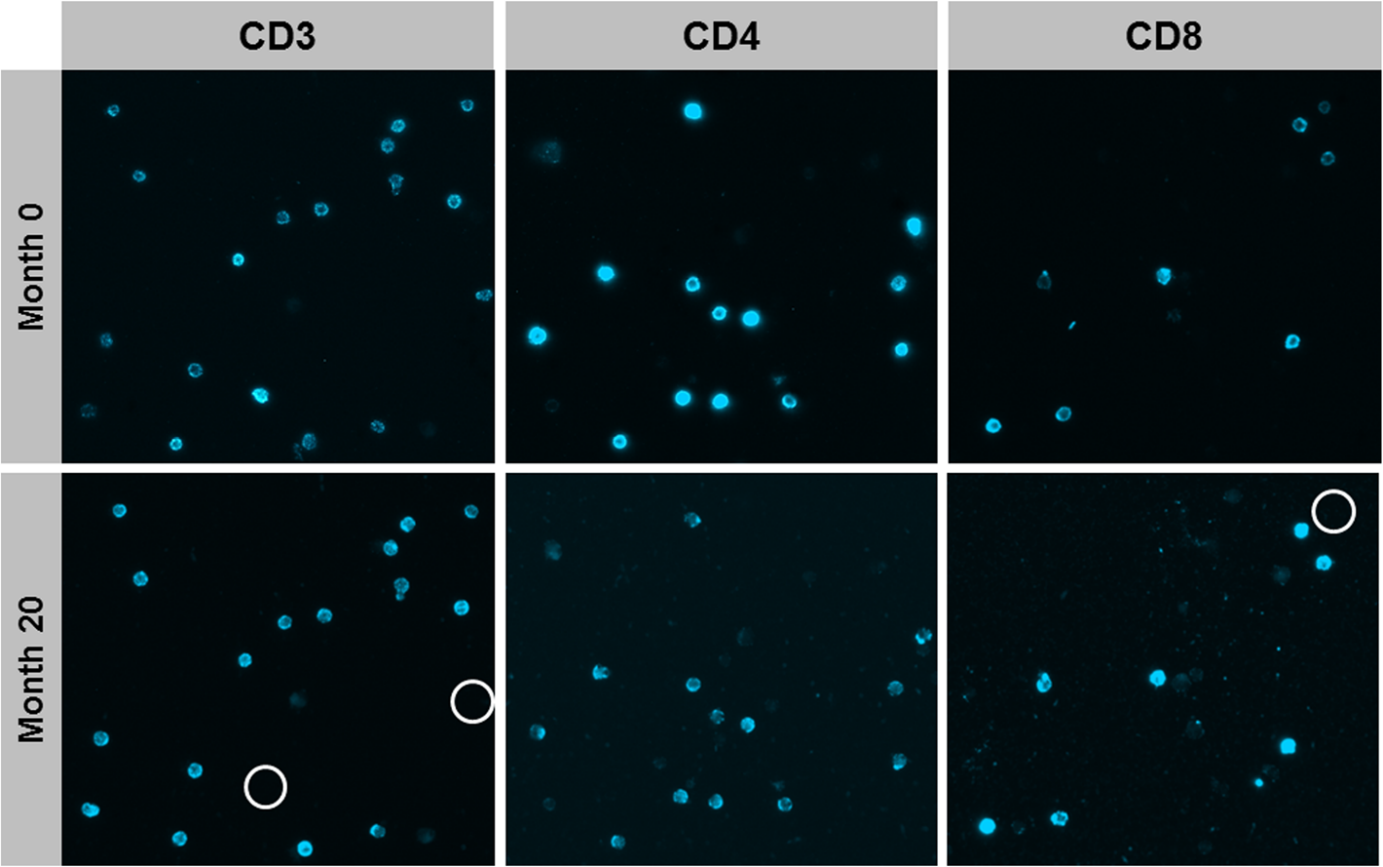
Raw fluorescence light images of CSF cells obtained from a patient with clinically isolated syndrome. Stainings and measurements were performed two times 20 months apart. White circles indicate cell loss

Long-term stability of samples is an essential prerequisite for the concept of iterative cytometry. We analyzed individual samples at different time points in order to assess whether changes in biomarker expression occur with prolonged storage. For long-term storage, the chip’s microfluidic channel was rinsed with PBS, sealed with plastic caps, and stored horizontally at 4 °C. Figure 3 shows an exemplary sample that was analyzed immediately after preparation and then reanalyzed 20 months later. The images demonstrate a high degree of biomarker stability but also reveal one main disadvantage of the method. As cells bind to the chip surface in a non-covalent manner, cell loss due to shearing forces may occur during staining and washing. We frequently observed a decreased cell density after repeated staining and washing steps. This raised concerns that artificial phenotypes might be generated if cells marked by automated cell recognition get lost during the procedure and therefore show zero expression in the following staining/imaging steps. We thus ran the cell recognition algorithm after completing all stainings of our panel. However, this procedure did not completely rule out the possibility of artificial phenotypes as cells would sometimes get detached during fluid exchange and then adhere to another position of the chip surface. Careful quality control was therefore carried out, and cells displaying artificial phenotypes were excluded manually.

### Cross-validation by flow cytometry

**Fig. 4:**
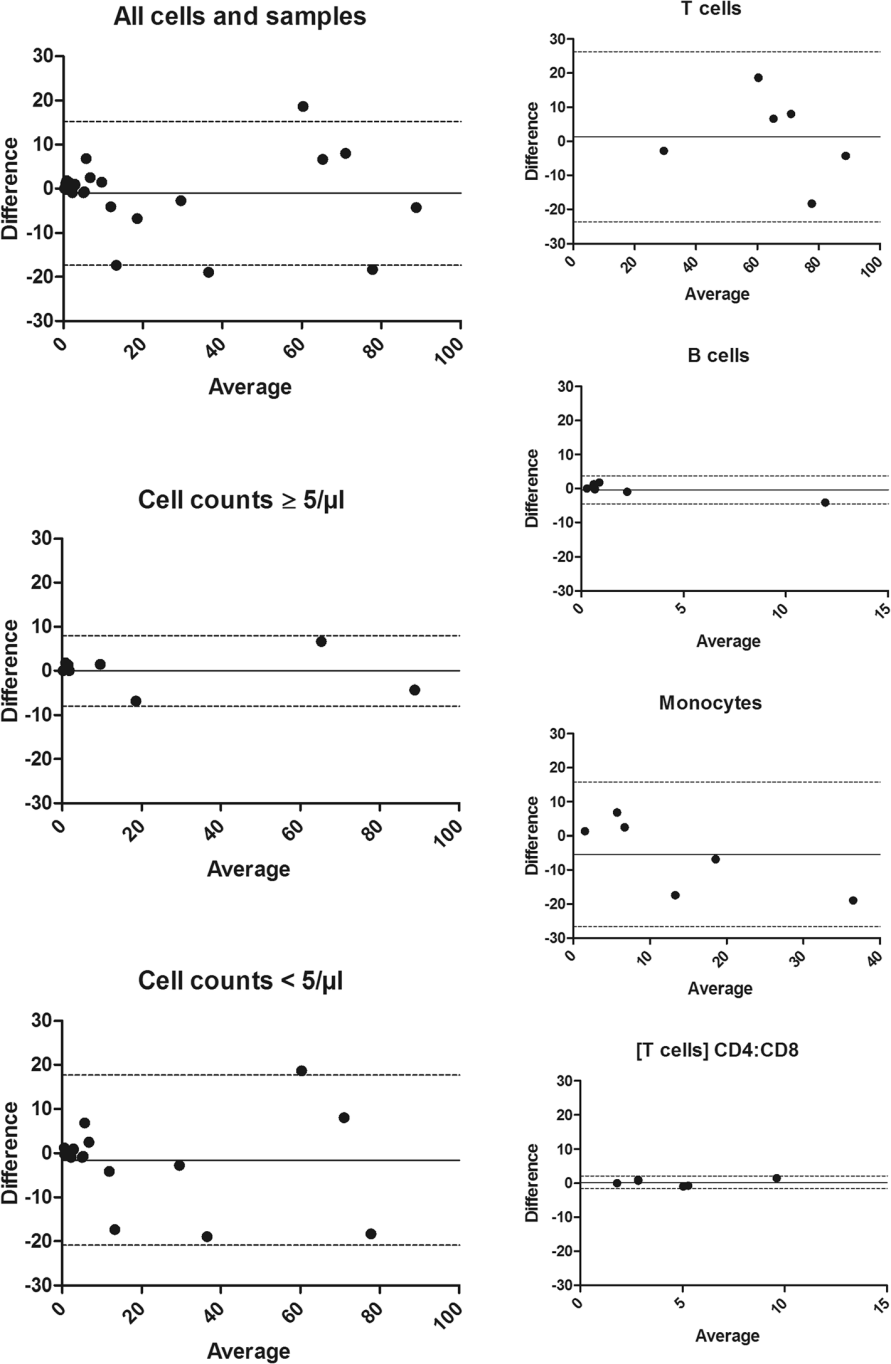
Bland-Altman plots comparing data obtained by Chipcytometry and flow cytometry. *X*-axes show average values of both measurements, *y*-axes show the difference between both values. Bias (continuous line) and 95% limits of agreement (dotted line) are indicated. All samples: *n* = 6, samples with cell counts ≥ 5/μl: *n* = 2

For cross-validation the Chipcytometry data were compared with results obtained by flow cytometry. The percentage distribution of distinct cell populations (CD3^+^ T cells, CD19^+^ B cells, CD14^+^ monocytes) and the T cell CD4:CD8 ratio were analyzed by both methods in the CSF of six patients. Agreement between the two methods was assessed by Bland-Altman analysis. We found a bias close to zero and a narrow 95% limit of agreement in samples with elevated cell counts but not in normocellular samples. Smaller biases and narrower limits of agreement were observed for B cells and CD4:CD8 ratio than for T cells and monocytes. The most pronounced bias (− 5.420) was found for monocytes which may indicate a tendency of Chipcytometry to measure higher monocyte percentages than flow cytometry (Table 4 and Fig. 4). Of note, in the analyzed cohort, there is one outlier for B cells, thus this result should be interpreted with caution.

**Table 4:** Bland-Altman analysis comparing data obtained by flow cytometry and Chipcytometry

Sample/cell population	Bias	95% limits of agreement	
All cells and samples	− 1.054	− 17.31	15.20
Cell counts ≥ 5/μl	0.008750	− 7.961	7.978
Cell counts < 5/μl	− 1.585	− 20.87	17.70
T cells	1.315	− 23.62	26.25
B cells	− 0.3833	− 4.463	3.696
Monocytes	− 5.420	− 26.62	15.78
T cell CD4:CD8 ratio	0.2733	− 1.638	2.185

A bias close to zero indicates that both methods generate nearly identical results

### Scientific application

**Fig. 5:**
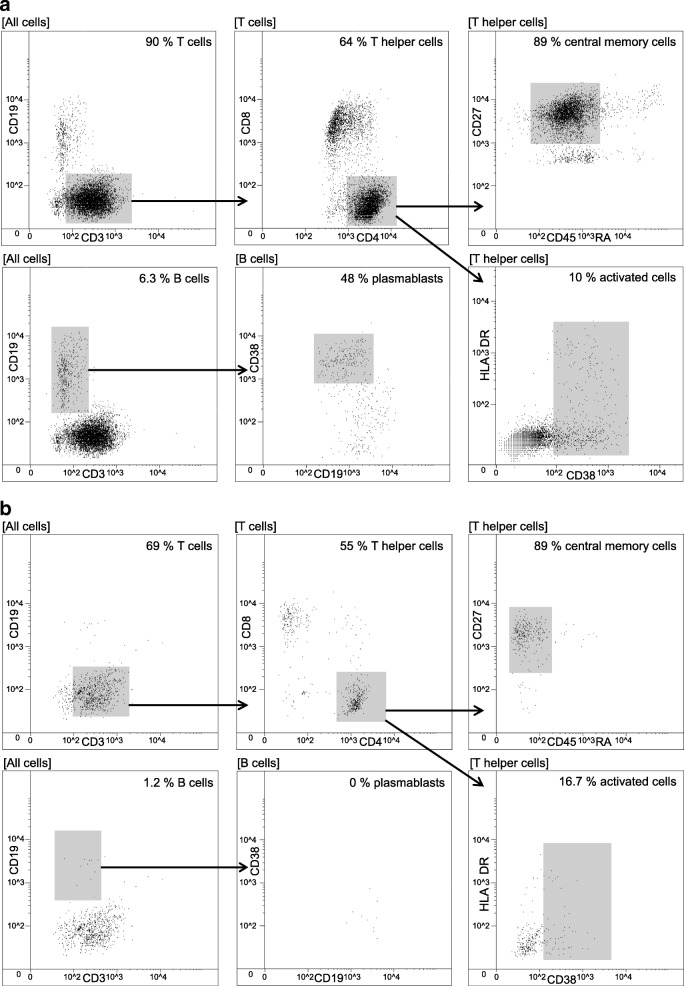
Chipcytometry analysis of CSF obtained from a patient with MS (**a**) and a patient with tension headache (**b**). Expansion of B cells and plasmablasts is evident in the CSF of the MS patient compared to the headache patient

We applied Chipcytometry to study immune cell subsets in the CSF of MS patients. Utilizing the potential of iterative cytometry a large amount of cellular biomarkers could be analyzed in each sample. In line with previous observations from flow cytometry studies [14, 15], the predominant cell population consisted of CD4^+^ T helper cells most of which had a central memory phenotype (CD27^+^ CD45RA^−^). Activation status of different T cell subsets was assessed by CD25, CD38, and HLA-DR staining. CD19^+^ B cells were frequent in the CSF which is a distinguishing feature of inflammatory central nervous system (CNS) disorders [16]. In line with published data [17], most B cells displayed a phenotype of class-switched memory cells (CD27^+^ IgG^+^) or plasmablasts (CD19^low^ CD27^+^ CD38^high^). Natural killer (NK) cells and monocytes appeared in the CSF in rather low frequencies. In addition, we constantly observed a population of lineage marker negative cells with strong HLA-DR expression indicating dendritic cells (DC) [18]. In summary, all relevant CSF cell populations could be easily detected, characterized, and quantified using Chipcytometry. Figure 5 shows exemplary data of one patient with MS and one control.

### Application in clinical practice

We report two cases in which Chipcytometry analysis of CSF cells proved useful in clinical routine.

#### Case 1

**Fig. 6:**
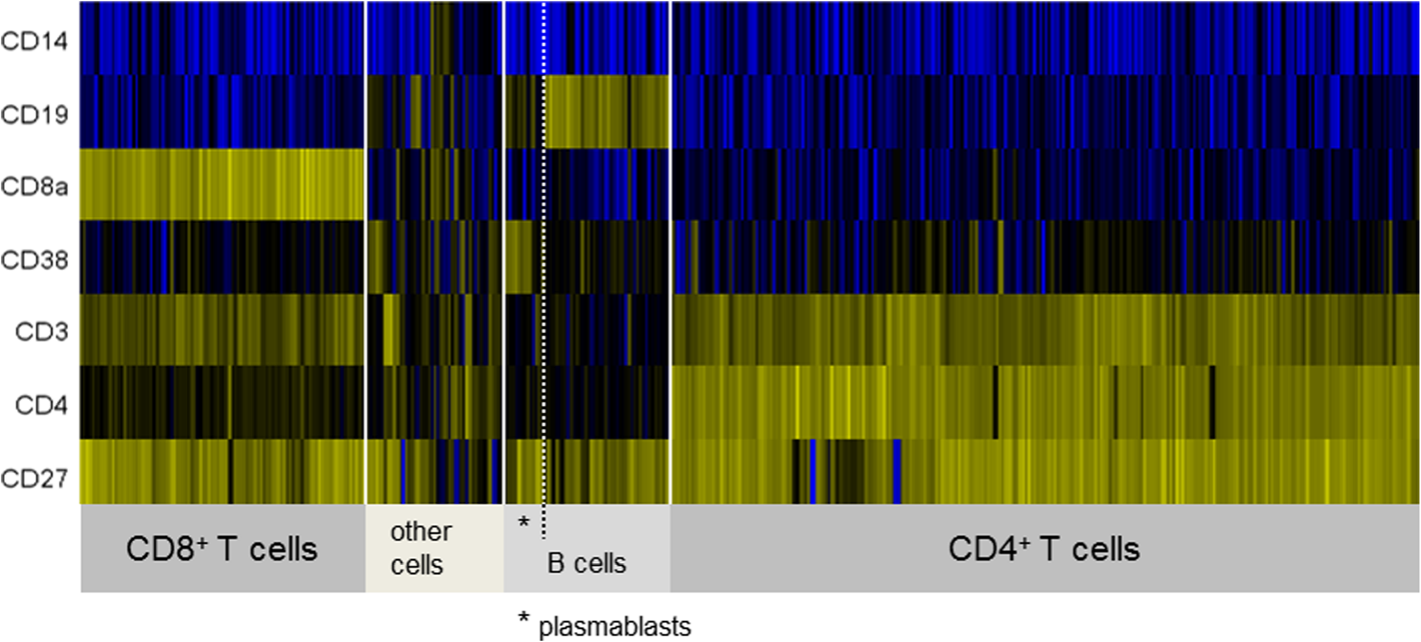
Chipcytometry analysis of CSF cells obtained from a patient with optic neuritis. Heatmap generated by cluster analysis. Note large population of B cells and plasmablasts

A 31-year-old woman with an unremarkable previous medical history was diagnosed with right-sided optic neuritis. MRI of brain and spinal cord showed multiple lesions with dissemination in space and in time so that a diagnosis of multiple sclerosis was made according to the McDonald criteria [19]. CSF cell count was 24/μl. Chipcytometry revealed 79% CD3^+^ T cells, 12% CD19^+^ B cells, and 1.5% CD14^+^ monocytes. T cell CD4:CD8 ratio was 2.7, B cell to monocyte ratio was 8.0. Fifteen percent of CD19^+^ B cells had a plasmablast phenotype (CD19^low^ CD27^+^ CD38^high^) (Fig. 6). A high B cell to monocyte ratio as in this case has been suggested to indicate rapid disease progression [2]. Immunomodulatory treatment was started immediately.

#### Case 2

**Fig. 7:**
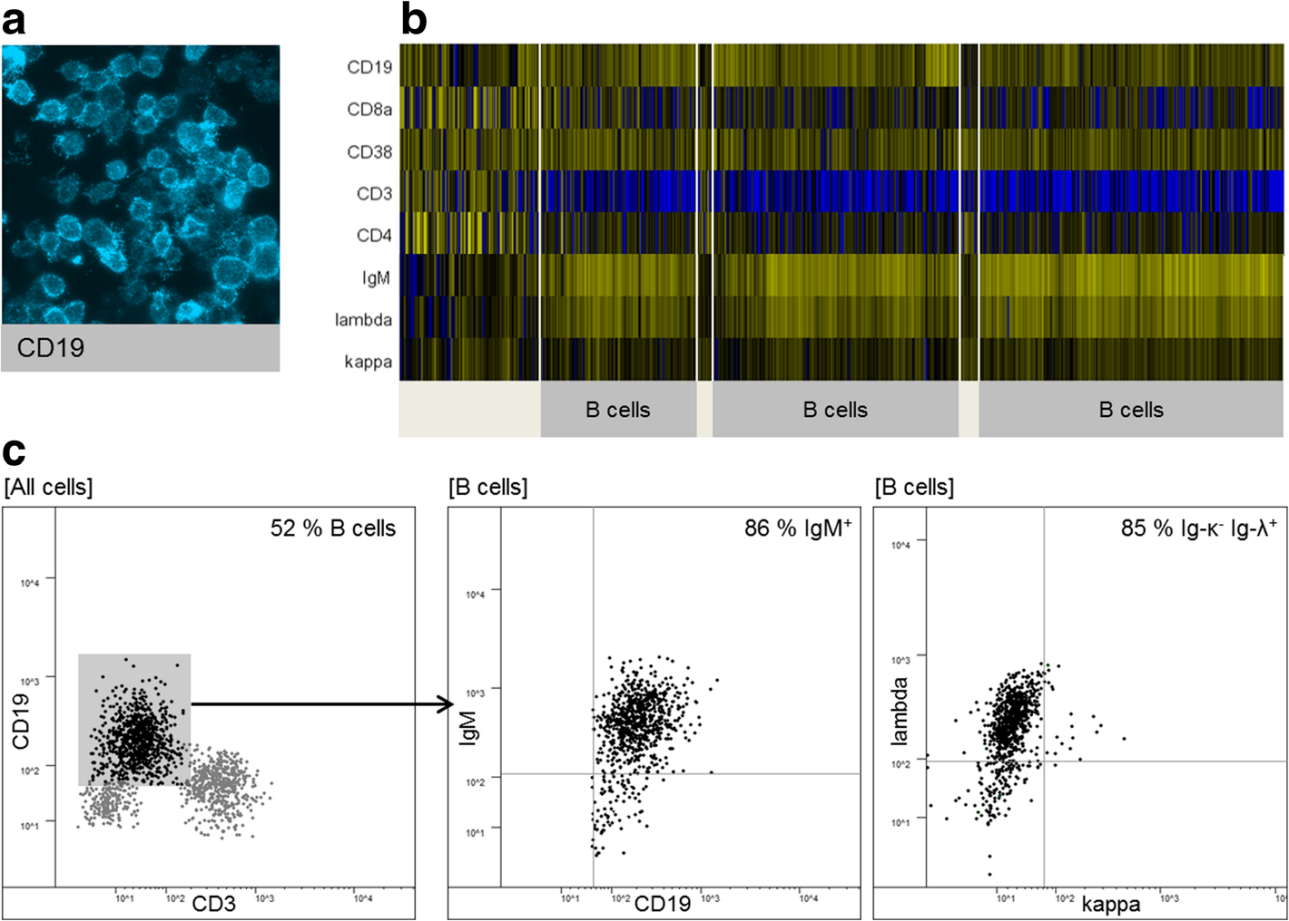
Chipcytometry analysis of CSF cells in a patient with lymphomatous meningitis. **a** Raw image (fluorescence light mode) of CD19-expressing B cells. **b** Heatmap showing a large fraction of B cells with uniform expression of IgM and λ-light chains. **c** 2D plot of CSF cells at a later date (lumbar puncture was repeated for intrathecal drug injections)

A 78-year-old woman suspected of having normal pressure hydrocephalus unexpectedly had an elevated CSF cell count of 80/μl. Our standard Chipcytometry panel (CD3, CD4, CD8, CD14, CD19, CD24, CD27, CD38, CD45RA, CD56, and HLA-DR) revealed a disproportionately high percentage of CD19^+^ B cells (84%) raising the suspicion of B cell lymphoma. We performed additional stainings with a set of B cell surface markers (IgA, IgD, IgM, IgG, κ-, and λ-light chains) which proved that B cells were IgM^+^ and had λ-light chain restriction (Fig. 7). A diagnosis of lymphomatous meningitis was made and intrathecal injections of methotrexate were started.

## Discussion

Immunophenotyping of CSF cells is a promising tool for diagnostic and scientific purposes. However, the invasive procedure required to collect CSF, its low cellular content, and the rapid decline of cell numbers ex vivo hamper conventional immunophenotyping by flow cytometry. The ideal method to analyze CSF cells would therefore require only small amounts of cells, be available immediately after sample collection, be able to measure multiple biomarkers in a single sample, and allow for long-term storage and reassessment of samples even at a different site. Chipcytometry was originally designed to address similar issues, albeit in the setting of pediatric patients with hematologic diseases [11]. As frequent blood withdrawal is stressful for severely ill children, a way to obtain as much information as possible from very small blood samples was sought. Chipcytometry has since proved useful in a number of clinical settings and with different sample materials [10–12].

In this work, we tested whether Chipcytometry is a suitable method for immunologic characterization of CSF cells. Our first observation was that the majority of CSF samples with normal cell counts < 5/μl could not be analyzed due to insufficient cell density on the chip surface. There are two main problems with low cell density. First, more positions (microscope fields of view) of the surface must be scanned to collect a sufficient number of cells. While the scanning procedure is rather quick, the bleaching step requires 30 s per position in the current setting. Therefore, lower cell densities account for increasingly time-consuming data acquisition. For 50 positions, bleaching alone would take 25 min in each staining-imaging-bleaching cycle. The second problem is that cell recognition depends on proper imaging, i.e., correct focus and exact repositioning in each cycle. These steps are fully automated but get increasingly error-prone when fewer cells per position are available. We aimed to increase cell yield by varying centrifuge settings, tube materials, and resuspension technique but did not achieve a relevant improvement. As a consequence, to achieve higher cell density either sample volumes must be increased or the surface area on which the cells are immobilized must be scaled down. In summary, in the current setting the amount of cells required is not sufficient for reliable analysis by Chipcytometry in the majority of normocellular CSF samples.

Another problem we encountered more frequently than expected was cell loss after staining or washing. Shearing forces on the chip surface are unavoidable during fluid exchange; however, we assume that suboptimal adhesive properties of CSF cells contribute to the problem as relevant cell loss was not reported in other Chipcytometry studies with different sample sources [9]. While specific cell adhesion molecules like LFA-1 and VLA-4 are generally overexpressed on CSF cells compared to peripheral blood [20] this is not likely to affect the non-selective binding to oligonucleotides involved in this method. On the contrary, the adverse characteristics of CSF that lead to rapid cell death ex vivo might also alter membrane properties so that adhesive forces are reduced.

Despite these drawbacks some definite advantages of Chipcytometry over conventional approaches can be determined. Preanalytic sample handling is quick and simple so that cells can be fixated on the chip within 40 min from lumbar puncture. Once fixated, the cells can be analyzed at a later time point or at multiple time points, also at a different site. We found excellent biomarker stability even up to 20 months after sample preparation (Fig. 3). There are different cell-stabilizing solutions available for flow cytometry, but none of these provide long-term biomarker stability. Furthermore, the possibility to measure a theoretically unlimited number of biomarkers in a single sample is a major advantage of this method. Importantly, it is not necessary to decide on a fixed set of markers beforehand. Establishing additional markers is possible at any time because no compensation steps are required as in multicolor flow cytometry. The case of our patient with unexpected lymphomatous meningitis illustrates how this increased flexibility can be highly useful in clinical diagnostics.

As there are several methodical differences between Chipcytometry and flow cytometry, cross-validation of the new method by the established standard is important. Our experiments showed a high grade of concordance between both methods in general. However, there were differences in regard to the cellularity of the samples and the analyzed cell populations. In samples with elevated cell counts, we found a better agreement between Chipcytometry and flow cytometry than in normocellular samples. It is to be expected that the error margin increases when fewer cells are detected. However, the results may also be explained by the fact that automated cell recognition is less accurate when cell density is low.

Both methods showed a very high grade of concordance when T cell CD4:CD8 ratios were analyzed. However, the results were slightly less congruent for T cell and monocyte fractions. Monocytes in particular appear to be slightly overestimated by Chipcytometry compared to flow cytometry. This may likely be explained by different gating strategies. In flow cytometry lymphocytes and monocytes are usually identified according to their light scattering properties. In our cross-validation experiments, we used forward scatter and CD45 for gating of leukocytes and then side scatter and CD14 to distinguish between lymphocytes, monocytes, and granulocytes. In Chipcytometry light scattering properties are not routinely measured, we thus relied only on lineage marker expression to identify cell populations. However, we cannot completely rule out the possibility that monocytes are positively selected during sample preparation because they might adhere stronger to the chip surface than lymphocytes.

## Conclusions

In summary, our results demonstrate the great potential of Chipcytometry of CSF cells for both scientific questions and routine diagnostic. However, the main problem with CSF cell analysis by Chipcytometry is the low amount of cells. The microfluidic chips used in this study were designed for peripheral blood which has a more than 1000-fold higher leukocyte concentration than CSF. We think that a new chip design optimized to meet the requirements of CSF, e.g., a reduced surface area with increased adhesive properties, would greatly enhance the value of this method. At the present time, however, Chipcytometry cannot be recommended for routine analysis of normocellular CSF samples hence flow cytometry remains the method of choice. Moreover, the results of cross-validation between flow cytometry and Chipcytometry need to be confirmed in a larger cohort, as the amount of six patients is a clear limitation of this study.

## Abbreviations

CIS: Clinical isolated syndrome
CNS: Central nervous system
CSF: Cerebrospinal fluid
DC: Dendritic cells
MFI: Mean fluorescence intensity
MRI: Magnetic resonance imaging
MS: Multiple sclerosis
NIND: Non-inflammatory neurological disease
NK cells: Natural killer cells
NND: Non-neurological disease
OIND: Other inflammatory neurological disease
PBMC: Peripheral blood mononuclear cells
PBS: Phosphate-buffered saline
PE: Phycoerythrin
PP: Polypropylene
PS: Polystyrene
